# The multidimensional relative poverty of rural older adults in China and the effect of the health poverty alleviation policy

**DOI:** 10.3389/fpubh.2022.793673

**Published:** 2022-07-22

**Authors:** Weihong Zeng, Pianpian Zhao, Yuan Zhao, Rashida Saddique

**Affiliations:** ^1^Jinhe Center for Economic Research, Center for Aging and Health Research, Xi'an Jiaotong University, Xi'an, China; ^2^Jinhe Center for Economic Research, Xi'an Jiaotong University, Xi'an, China

**Keywords:** relative poverty, multidimensional poverty, health poverty alleviation policy, older adults, treatment effect model

## Abstract

**Introduction:**

Although, especially in the past decade, poverty measurement approaches have been duly developed in two paths (from unidimensional to multidimensional poverty and from absolute to relative poverty), merely a few studies have focused on the combination of both perspectives. However, with global aging, poverty among older adults simultaneously presents multidimensionality and relativity characteristics. This paper explores a multidimensional relative poverty index (MRPI) relative to the aged group in four dimensions, namely, health, social, mental, and material, and then empirically evaluates the specific effects on the MRPI of one of the key targeted anti-poverty policies, that is, the health poverty alleviation policy (HPAP), which includes public health service, medical expense reimbursement, rewarding assistance, basic medical insurance, and so on.

**Methods:**

Using pooled cross-sectional data of poverty alleviation from 2014 to 2020 with a total of 83,521 observations aged 60+ in County J, Shaanxi Province in China, we calculate the MRPI for the older adults *via* a fuzzy set approach. Statistical difference testing is used to analyze the characteristics and trends of the MRPI. In policy evaluation, to address endogenous problems, the treatment effect model based on Heckman's two-stage regression and finite distributed lag model are used with a controlled township cluster structure.

**Results:**

From 2014 to 2020, the MRPI shows a significant upward trend for older adults in rural China, and the health component takes the dominant MRPI position. Empirically, we find that the HPAP can significantly alleviate the MRPI of older adults. Furthermore, among the health poverty alleviation measures, basic medical insurance is the most effective anti-poverty policy to support older adults. Specifically, empirical evidence shows that there is a more statistically significant reduction in the MRPI with the HPAP for the sub-group of older adults with chronic diseases or disabilities.

**Conclusion:**

Both relativity and multidimensionality should be emphasized when analyzing poverty *vis-à-vis* the aging society, and for this, the MRPI is one of the effective tools. Comparing the relativity with the aged group engenders a more accurate understanding of their poverty situation. Moreover, the importance of the health component among all the four dimensions is more conducive to the detailed analysis of their poverty. The empirical analysis results show that regarding poverty reduction approaches in China, developing integrated health promotion systems is necessary and imminent, especially in the long run, such as long-term care insurance that covers typical disabled older adults with chronic diseases.

## Introduction

In the past decade, several theoretical analyses on the nature of poverty have been conducted, and a vast empirical literature has been produced in two branches. One is developed from unidimensional to multidimensional poverty (1–7), while the other is expanded from absolute to relative poverty (8–11). Only a few studies conceptually touch topics bordering on combining both perspectives (12, 13). This paper presents an attempt at this combination by constructing a multidimensional relative poverty index (MRPI) to measure the poverty levels among older adults.

From a life cycle perspective, poverty at old age is an important component that affects people's life-long wellbeing, which cannot be ignored (14). Compared with others, older adults are a special and vulnerable group because they can easily fall into poverty and find it challenging to extricate themselves from penury through their individual efforts, such as employment (15, 16). Along with age, it is inevitable for older adults to face the increasing health risks of functional limitation and medical expenditure for chronic diseases. Meanwhile, their ability to learn, adapt, and participate in society gradually decreases (17, 18).

On the one hand, poverty measured by income or consumption as a unidimensional standard is likely to overestimate the poverty ratio for the aged group (18–20). Generally, the standard is determined by the average level of the entire population, although the income or consumption of seniors hovers around the lower level relative to the entire population. According to Sen's “capability approach,” low-monetary income cannot wholly explain poverty, while deprivation in other dimensions is what causes it (1, 21–24). Based on the extensive literature (16, 25–27), poverty among older adults emerges in four dimensions. Materially, they are mainly supported by fixed pensions and intergenerational support, which make resisting the impact of external risks challenging (28–30). Regarding the health dimension, chronic, sudden, or serious diseases are generally accompanied, and their physical health is generally lower than that of other groups (31). Mentally, “empty nest” older adults are dramatically increasing, especially with the population transformation of less children and family structure change, and the mental health problem among older people is becoming increasingly prominent (16). Socially, with the rapid development of social media and digital economics, the social networks of the old adults are further compressed, and the seniors are said to be “digital refugees” (32, 33).

On the other hand, differing from those of the younger age groups, the use of the absolute poverty line neither reflects the true situation of older people nor is it indicative of the vulnerability and depth of poverty (2, 23, 34–36). Rather than absolute poverty, relative poverty refers to the poverty state of a reference object. It is generally expressed by the relative deprivation of a certain situation of an individual from the highest one. And it is dynamic from a life cycle standpoint. However, there is no consensus on how to determine the relative poverty standard (37). In general, the understanding of relativity entails to two terms: relative to the entire population and relative to the older population. Relevant studies only focus on the former rather than the latter. However, as mentioned above, older adults have evident vulnerabilities compared to other populations, mainly in terms of material, mental, social, and health. When comparing older adults to the entire population, it is clear that older adults are poor in many dimensions. Considering the similar cohort characteristics, we believe the study of individual relative poverty compared with the old group itself will provide a better understanding of the poverty level and characteristics of older adults.

Thus, in the second part of this paper, we construct the MRPI for older adults, combined with the four dimensions of material, health, mental, and social, and use the population aged 60+ as a frame of reference. Thereafter, we decompose the index to determine the importance of the different dimensions.

Over the past decade, the Chinese government has made great efforts to reduce poverty on a large scale. One of the anti-poverty policy innovations entails that different policies are designed for various causes of poverty, an approach that is referred to as the targeted poverty alleviation policy. To minimize the risk and cost associated with the health problem, a complete security system was established to ensure accessibility to basic medical and health services in 2016. This strategy includes new rural cooperative medicine, serious illness insurance, medical assistance, and supplementary medical insurance (38). Subsequently, in 2017, the categorization and treatment of poor people suffering from serious illnesses and chronic diseases further facilitated the implementation of health assistance packages for poor people. Additionally, targeting the poor due to their health problems specifically, a wide range of preferential policies, institutional arrangements, and support measures, including public health services, reimbursement of medical expenses, and incentive assistance was implemented. From the current practice results, these targeted poverty alleviation policies have indeed played a role in poverty alleviation (39, 40). In 2020, absolute income poverty was eliminated based on the World Bank's current standard of US$ 1.9 per person per day. According to the National Bureau of Statistics, 98.99 million rural poor people have been lifted out of absolute income poverty (41).

However, from the perspective of multidimensional relative poverty (MRP), the evaluation of these targeted policies to determine the effects for the specific dimension of relative poverty is significant, especially for older adults, who face much higher health risks and potential medical expenses in the long term as they age. Therefore, in the third part of this paper, we evaluate the poverty alleviation policy implemented for rural older adults.

The remainder of this paper proceeds as follows: Section 2 describes the measurement of the MRPI for older adults in China. Section 3 details the empirical evaluation of the health poverty alleviation policy (HPAP) on the MPRI. Section 4 discusses the results. Section 5 presents the conclusion and policy suggestions.

## Measurement of the MRPI for rural older adults

### Data source

The data are obtained from the “Poverty Alleviation Database” in County J in Shaanxi Province, which has been constructed by the Chinese government for all poverty households since 2014. The identification of poverty households is based on multidimensional poverty indicators. Specifically, the whole household is identified based on the household income, housing, education, health, and other conditions. Once the application meets the identification criteria, the household's information is entered into the database, and the data are reviewed and updated regularly. The database structurally covers the individual demographic information, household structure, health status, living standard, income level, and poverty intervention policy involved.

County J in Shaanxi Province is a typical poverty-stricken county in Northwest China. In 2014, 15.52% of the population was living under the absolute income poverty line in County J. After the poverty alleviation intervention, up to February 2020, County J successfully achieved the exit criteria for the poor.^1^ County J comprises 13 townships, each of which has a different population size and economic status. The distribution of the total sample and older adults' sample in County J from 2014 to 2020 is shown in Table 1. The total sample population in the database is 318,220, including 83,521 old adults aged 60+, accounting for more than one-quarter of the total sample.

**Table 1:** The total sample and the older adults' sample size from 2014 to 2020.

**Year**	**Total sample**	**Older adult sample**	**%**
2014	41,365	9,877	23.88%
2015	40,934	10,335	25.25%
2016	48,168	9,931	20.62%
2017	48,379	10,306	21.30%
2018	47,290	14,180	29.99%
2019	46,402	14,341	30.91%
2020	45,682	14,551	31.85%
Total	318,220	83,521	26.25%

### Selection of dimensions

As mentioned above, we construct the MRPI in four dimensions: health, social, mental, and material (16, 25–27). Table 2 presents the detailed dimensions and indicators with the selected variable descriptions.

**Table 2:** Compositions of multidimensional poverty dimensions and indicators for older adults.

**Dimensions**	**Primary indicators**	**Secondary indicators**
Dim_1_: Health	Health Status	Disability: Presents at least one disability = 1, otherwise 0.
		Disease: Suffers from at least one disease = 1, otherwise 0.
	Health Insurance	No commercial health insurance = 1, otherwise 0.
Dim_2_: Social	Social Participation	No work: No work = 1, otherwise 0.
	Social Security	No basic pension = 1, otherwise 0.
	Sources of Information	No radio/TV at home = 1, otherwise 0.
	Political Participation	Not a party member = 1, otherwise 0.
Dim_3_: Mental	Adapt Ability	Education level: 0 illiteracy; 1 primary school; 2 junior high school; 3 high school; 4 professional training college; 5 bachelor's degree and above.
	Sense of Loneliness	Live alone: living alone = 1, otherwise 0.
Dim_4_: Material	Income	Per capita income of the old adults in RMB yuan.
	Living standards	Housing area per capita in squared meters.
		Non-clean fuel: Non-clean fuels at home= 1, otherwise 0.
		No electricity at home = 1, otherwise 0.
		No safe drinking water at home = 1, otherwise 0.
		No sanitary toilet at home = 1, otherwise 0.

#### Health dimension

This includes health status and health insurance. Health status is measured by whether the older adult is disabled or with at least one type of disease (42). Owing to the full coverage of basic public medical insurance available now, health insurance here refers to commercial supplementary health insurance (43).

#### Social dimension

This includes social participation, social security, information sources, and political participation (44–46). Social participation is measured by still outworking or not; social security is measured by holding a basic pension or not; the information sources in rural areas for older adults are mainly radio or TV, so we measure information accessibility by possessing a radio/TV or not; political participation is also an important aspect of the social dimension, and we measure the political participation of the seniors by being party members or not.

#### Mental dimension

This includes learning, adaptive ability, and loneliness (30). Because of the unavailability of the learning and adaptive ability information in the data set, the proxy indicator of educational attainment is chosen. The higher the educational level of the older adult, the higher his or her learning and adaptive abilities (47). Additionally, living alone is used to measure the loneliness of the older adults.

#### Material dimension

This includes income level and living standard. Income level is a continuous variable measured as per capita income. The living standard refers to the basic living condition with housing area per capita, clean energy, electricity, safe drinking water, and sanitary toilet. Housing area per capita is a continuous variable measured in squared meters. If clean fuel, electricity, safe drinking water, or sanitary toilet are not available to the older people, the indicator value is 1. This means that they have a poor standard of living conditions, otherwise it is 0.

Appendix A presents the statistics of all the indicators for the four dimensions by year. It can be seen that in the health dimension, ~13.6% (46.5%) of the older adults are disabled (suffer from diseases). Very few seniors have medical supplemental insurance except public health insurance. All the social dimension indicators show an upward trend. In 2014, only 1.5% of seniors are employed, 95% have pension insurance, 1.7% are party members, and 20.8% have a radio/TV at home. Moreover, up to 2020, the ratios rise to 15.4, 100, 5.1, and 73.1%, respectively. For the mental dimension, an upward education level trend of the older adults is also shown. The rate of living alone in the older people decreases from 30.7% in 2014 to 23.3% in 2020. With the rapid aging phenomenon in rural areas in recent years, the oldest-old group of seniors is increasing, the cases of living with informal caregivers are slightly increased, thereby elucidating why the case of living alone decreases. Materially, the housing area of the older adults changed a little overall, but the income increased significantly. Notwithstanding the full coverage of electricity in 2016 and safe drinking water in 2018, only a few older adults started to use clean energy in 2020, and approximately half of them had sanitary toilets in their houses.

### Construction of the MRPI

Owing to the emphasis on the relativity of multidimensional poverty with a large sample size in our study, we apply the fuzzy set approach in calculating the MRPI. Compared with the popular AF method developed by Alkire and Foster (4, 5, 48, 49), which is widely used in studies on absolute multidimensional poverty, the fuzzy set approach can not only measure relative deprivation but also raise the data-driven endogenous weight, which reflects the relative importance of certain indicators (50–54).

The fuzzy set approach replaces the criterion of poverty line with poverty under a range of segments (7). Cerioli and Zani (55) constructed a fuzzy theoretical model for the multidimensional analysis of poverty, which was later developed and improved by Cheli and Lemmi (50), resulting in the totally fuzzy and relative method. It is based on the membership degree functions to obtain the indicators of deprivation given sample variables, and the values obtained from it are used to reflect the relative degree of deprivation of individuals. Thus far, the fuzzy set approach has been widely applied in measuring multidimensional poverty (50–54). Meanwhile, another advantage of this method is that the calculation of weight depends on the intensity of the relative deprivation in different dimensions, which overcomes the deficiency of equal weight in the calculation of multidimensional indicators.

The MPRI calculation and its decomposition are based on the following four steps.

#### Step 1: Determining the membership degree function

We classify the above-mentioned indicators into three types, namely, binary, discrete, or continuous variables. Thereafter, we determine their membership degree functions (56).

1. *Binary variables*. For binary indicators, if the membership degree function μ_*p*_ is defined to have a value of 1, the possession of the item is assumed to be more prone to poverty for the older adult. Otherwise, it is 0.

$$\begin{array}{l} \mu_{p}=\{\begin{array}{c} 1,\;x_{ij}=1\\ 0,\;x_{ij}=0 \end{array} \end{array}$$

where, *x*_*ij*_ represents the value of the *i*th person on the *j*th indicator, *i* = 1,2,.... *n*; *j* = 1,2,... *k*. As shown in Table 2, we define the values of Disability, Disease, No health insurance, No work, No pension, No radio or TV, Not a party member, Non-clean fuel, No electricity, No safe drinking water, No sanitary toilet, and Live alone, as the indicators of membership, as 1.

2. *Discrete or continuous variables*. Discrete or continuous variables can only have one possible value within a certain range, such as Education level, Income, and Housing area indicators. We define the membership degree function μ_*p*_ as (50):

$$\begin{array}{l} \mu_{p}=\{\begin{array}{c} 0\;x_{ij}\geq x_{\max,j}\\ \frac{x_{\max,j}-x_{ij}}{x_{\max,j}-x_{\min,j}}\;x_{\min,j}<x_{ij}<x_{\max,j}\\ 1\;x_{ij}\leq x_{\min,j} \end{array} \end{array}$$

where *x*_min,*j*_ and *x*_max,*j*_ denote the minimum and maximum values of the *j*th indicator except the outliers, respectively. The further away the value of *x*_*ij*_ is from *x*_max,*j*_, the more likely the individual is to be relatively poor. For instance, for the education level, *x*_min,*j*_ represents illiteracy, and if the education level of an older adult typifies illiteracy, the individual is considered to be extremely poor based on this indicator, that is 1. Similarly, for income and house area, when the indicator value is equal to *x*_max,*j*_, the membership degree is 0, which means that the older people are exempt from any risk of poor on this indicator.

#### Step 2: Determination of weights

Based on Cheli and Lemmi's (50) method, higher weights should be given to indicators that can easily lead to relative poverty. For example, if the indicator “no safe drinking water” is equal to 1, then give it a higher weight. This is an endogenous weighting method. Using endogenous weights with one particular dataset can reflect the importance of the different indicators in the composite measure (57, 58). Endogenous weight overcomes the shortcomings of the traditional equal weight method, which is arbitrary and varies with the number of indicators (49).

The weight for the indicators is calculated as:


$$\begin{array}{l} w_{j}=\ln\left[\frac{1}{\frac{1}{n}\sum_{1}^{n}\mu_{p}\left(x_{ij}\right)}\right] \end{array}$$

where μ_*p*_(*x*_*ij*_) denotes the membership value of the *i*th older adults on the *j*th indicator, and *n* is the sample size. The endogenous weight calculated for each indicator over the years is shown in Appendix A.

#### Step 3: Calculating the MRPI

Combining the membership degree function and the weight, the MRPI for each old adult is calculated as


$$\begin{array}{l} MRPI_{i}=\frac{\sum_{j=1}^{k}\mu_{p}{\left(x_{ij}\right)}^{*}w_{j}}{\sum_{j=1}^{k}w_{j}} \end{array}$$

where μ_*p*_(*x*_*ij*_) denotes the membership value of the *i*th individual on the *j*th indicator, *w*_*j*_ is the weight of the *j*th indicator, and κ is the number of the indicators.

Table 3 shows the average MRPI for older adults over the years and the group difference. It can be seen that MRPI showed an upward trend from 2014 to 2019, except for the sudden decline in 2020 to achieve the poverty eradication goal. Compared to 2014, the MRPI jumped from 0.124 to 0.350 in 2019. This result is contrary to the absolute multidimensional poverty trend among older adults: it shows a decreased trend from 0.394 in 2014 to 0.289 in 2016 (59). A possible reason for the significant rise in relative poverty among the older adults in 2019 is the time lag of other poverty alleviation policies. For example, albeit the industrial poverty alleviation policy intervened heavily in 2018, the effect of poverty alleviation can be realized only after a year, such as planting and farming. Additionally, the industrial poverty alleviation policy mainly targets young older people who have the ability to work, and the data show that 56.68% of the older people who participate in industrial poverty alleviation are under 70 years old, which further divides the relative poverty of the older adults.

**Table 3:** The MRPI for rural older adults from 2014 to 2020 and group difference.

**Year**	**MRPI**	**Gender**		**Adjusted**	**Age Groups**			**Adjusted**	**Household**		**Adjusted**
		**Female**	**Male**	**Wald test**	**Group 1**	**Group 2**	**Group 3**	**Wald test**	**Non-poor**	**Poor**	**Wald test**
					**Aged 60**+	**Aged 70**+	**Aged 80**+				
2014	0.124	0.126	0.123	^*^	0.119	0.127	0.146	^***^	0.085	0.126	^***^
2015	0.159	0.161	0.157	^**^	0.151	0.163	0.185	^***^	0.134	0.166	^***^
2016	0.190	0.193	0.187	^**^	0.175	0.199	0.236	^***^	0.150	0.206	^***^
2017	0.166	0.168	0.165		0.154	0.173	0.201	^***^	0.136	0.181	^***^
2018	0.201	0.203	0.200		0.187	0.210	0.236	^***^	0.185	0.243	^***^
2019	0.350	0.357	0.343	^***^	0.324	0.375	0.392	^***^	0.338	0.489	^***^
2020	0.141	0.144	0.138	^***^	0.130	0.151	0.160	^***^	0.141	-	-

*** *p < 0.01*,

** *p < 0.05*,

* *p < 0.1*.

Gender difference exists, showing that the MRPI is significantly higher for females than for males. This is because female elderly health is at a disadvantage compared to male health, both in terms of physical and mental health (60, 61). Rural poor older females lack resources and opportunities, both socially and economically, and are a vulnerable group with a low-survival capacity (62).

The MRPI of the older age group 3 aged 80+ is higher than that of younger age group 1 aged 60+. The MRPI of the older adults in the poor household group is higher than that of the non-poor, and this maintains its statistical significance over the years. This suggests an identification divergence between unidimensional and multidimensional poverty, a situation that can be explained by the fact that while income is important, multidimensional poverty measures (including this relative standard) reveal dimensions of poverty that cannot be reached by increasing household income (11).

Thus, we can conclude that females in the oldest age group and living in poor households will be the most likely to experience MRP.

#### Step 4: Decomposition of the MRPI

To appraise its relative importance based on the contribution rate for different dimensions, we decompose the MRPI. Take the first health dimension as an example, it is calculated as:


$$\begin{array}{l} Contribution\text{\_}Rate_{Dim_{1}}=\frac{\sum_{j=1}^{3}\mu_{p}{\left(x_{ij}\right)}^{*}w_{j}}{\sum_{j=1}^{k}w_{j}} \end{array}$$

**Figure 1:**
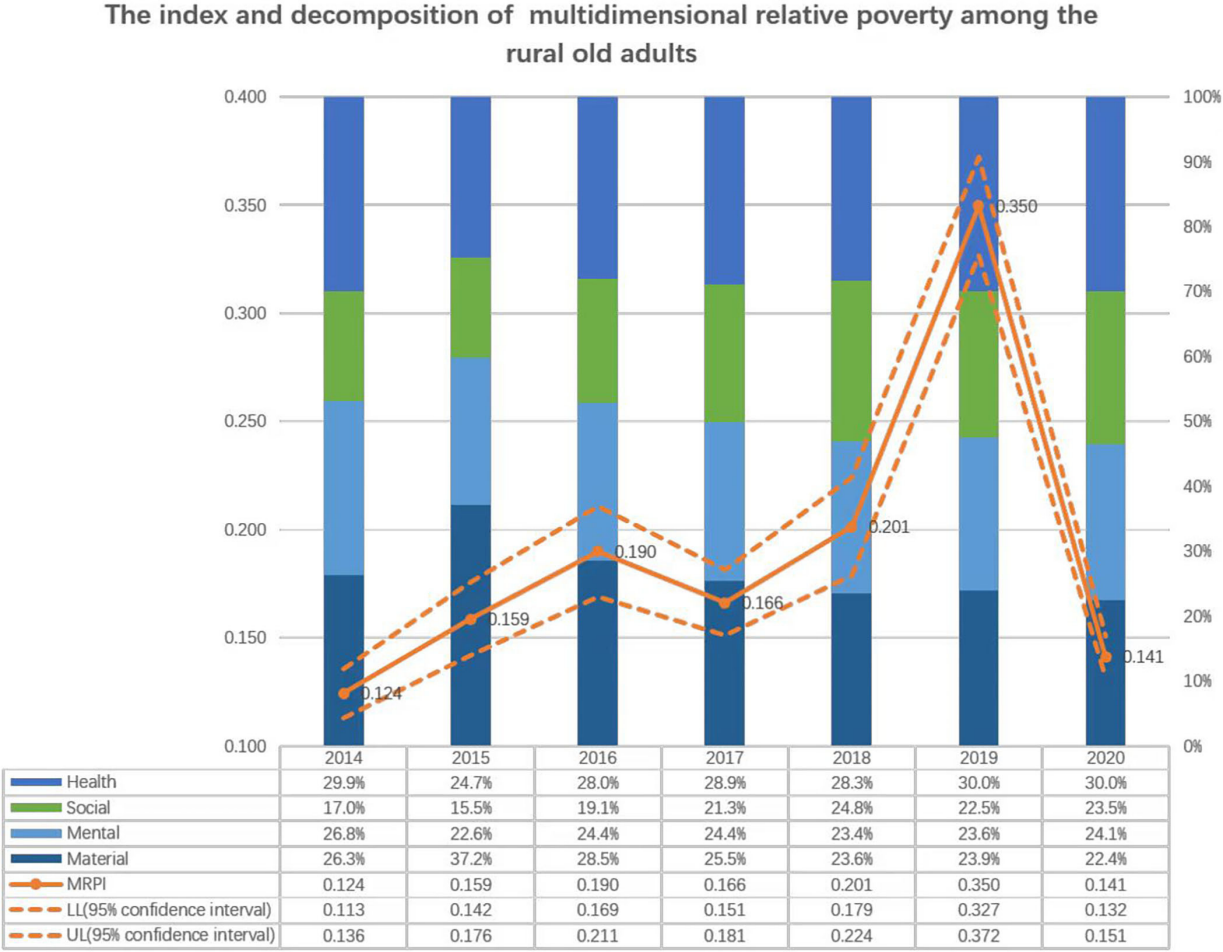
The index and decomposition of multi-dimensional relative poverty among the rural old adults.

Figure 1 shows the MRPI and the contribution rate for the four dimensions. It shows that among the four dimensions, the health component has the highest contribution rate to the MRPI for older adults. It maintains a stability of 29.9% in 2014 and 30.0% by 2020. That is, approximately one-third of the MRPI determinants for older adults is the health dimension. Conversely, the contribution rate of the material dimension showed an overall decreasing trend, and in 2020, it was the lowest, accounting for only 22.4%. This is consistent with the declining facts of material demand relative to other dimensions under the condition of rapid economic development, especially for older adults.

Therefore, the health dimension is the most important composition that cannot be ignored when analyzing poverty among older adults (42). In 2014, 49% of the older adults were under the poverty line owing to diseases, and the proportion increased to 55.3% in 2016. In 2016, an important targeted poverty alleviation policy, named the HPAP, began to be implemented, and, consequently, the proportion of the poor older adults caused by diseases fell from 45.7% in 2017 to 37.1% in 2020.

Does this targeted policy intervention effectively affect the MRPI for older adults? In the following part, we check the determinants of the MRPI *via* ordinary least squares (OLS) as a benchmark model and then evaluate the effect of the HPAP on the MRPI. Furthermore, for the analysis of heterogeneity, this paper examines which health poverty alleviation measure is the most effective, as well as which subgroup of people benefits the most.

## Determinants of the MRPI and evaluation of the HPAP on the MRPI

### Benchmark model for the determinants of the MRPI

We apply OLS for analyzing the determinants of the MRPI as a benchmark model^2^:


$$\begin{array}{l} Y_{it}=a_{it}+\alpha X_{it}+\delta_{i}+\mu_{it} \end{array}$$

where,

*Y*_*it*_: MRPI for older adult *i* at time *t*.

*X*_*it*_: the control variables, including the demographic characteristics of an individual: gender, age, and ability to work; family characteristics: if in a poor household or not, family size, the number of patients, the number of children, and the number of students in the family; and the natural assets: the area of cultivated land per person and the area of fruit land per person.

δ_*i*_: the township and year fixed effects.

μ_*it*_: error term.

Although the process of identifying poor households is based on certain criteria, the poverty status varies widely across townships. Therefore, in the following analyses, we control the township cluster for the survey data.

### Treatment effect model for the evaluation of the HPAP on the MRPI

Owing to the voluntary nature of applying for the support of the HPAP among the poor old adults, self-selection bias occurs. To investigate the possible effects of participating in health poverty alleviation on the MRPI of the old adults, we use Heckman's two-stage regression with the treatment effect model to evaluate the policy effect (63–66).

Regression model (second stage):


$$\begin{array}{l} Y_{it}=a_{it}+\beta I_{it}+\alpha X_{it}+\delta_{i}+\epsilon_{it} \end{array}$$

Selection model (first stage):


$$\begin{array}{l} I_{it}=\gamma Z_{it}+\rho X_{it}^{{\;}^{\prime }}+v_{it}\\ Prob\left(I_{it}=1|Z_{it}\right)=\Phi \left(\gamma Z_{it}\right) \end{array}$$

where in the first- and second-stage regression model:

*I*_*it*_: dummy variable, HPAP = 1 indicates that the old adult involved in the intervention of the health poverty alleviation policy, otherwise, it is 0;

$X_{it}^{{\;}^{\prime }}$: Except for the control variables in the benchmark model, we add the policy intervention variables, including social security guarantees, farmers' cooperatives, industrial poverty alleviation, and relocation of migrants, to control for the policy binding effect. To control the endogeneity of self-participation of these policies, the lag phases of the policy are controlled.

In the selection model for the first stage:

*Z*_*it*_: instrumental variables (IVs). Based on the research of Schultz and Yang (67–69), we evaluate health care access for older adults by the distance away from the main road as instruments. It is reasonable to assume that the farther the distance from the main road is, the higher the cost of participating in the health poverty policy is, such as information and transportation cost. Therefore, it lowers the probability of HPAP participation. In addition, poor villages have poor medical facilities and poorer medical standards compared to non-poor villages; thus, their demand for HPAP participation is higher. We selected if the old people live in a poor village as an IV.

### Variable statistics

Table 4 provides the description and summary statistics of the selected variables.

**Table 4:** Descriptive statistics for selected variables, 2014–2020.

**Variable**	**Total sample**				**HPAP** = **0**	**HPAP** = **1**	**Difference**
	**Mean**	**Std. Err**	**Min**	**Max**	**Mean**	**Mean**	***t*** **-test**
**MRPI**	0.194	0.006	0.011	0.996	0.148	0.232	^***^
**Characteristics of the older adults**							
Gender (Male = 1)	0.509	0.007	0	1	0.52	0.499	^***^
Age	70.561	0.227	60	106	70.271	70.806	^***^
Ability to work	0.141	0.015	0	1	0.141	0.141	
**Family characteristics**							
Poor household	0.453	0.032	0	1	0.648	0.289	^***^
Family size	2.141	0.063	1	9	2.035	2.23	^***^
Number of patients	1.075	0.035	0	8	0.996	1.141	^***^
Number of children	0.097	0.012	0	5	0.082	0.11	^***^
Number of students	0.147	0.017	0	5	0.118	0.172	^***^
Cultivated land	1.540	0.084	0	4.852	1.328	1.718	^***^
Fruit land	0.093	0.034	0	3.892	0.112	0.077	^***^
**Intervention policies**							
HPAP	0.543	0.018	0	1	-	-	-
Social security guarantees	0.341	0.039	0	1	0.328	0.352	^***^
Farmers' cooperatives	0.490	0.020	0	1	0.196	0.738	^***^
Industrial poverty alleviation	0.216	0.012	0	1	0.026	0.376	^***^
Relocation of migrants	0.004	0.001	0	1	0.002	0.005	^***^
**Instrumental variables**							
Distance	0.411	0.046	0	50	0.436	0.39	^***^
Poor village	0.191	0.022	0	1	0.166	0.212	^***^
**Sample size**		83,350			37,814	45,536	

*** *p < 0.01*.

A total of 83,350 older people are observed, and 54.26% of them are involved in health poverty alleviation program. The statistical difference in the MRPI is noted between the older adults who do not participate in the HPAP (MRPI = 0.148) and those who do (MRPI = 0.232), suggesting that the older adults who participate have a worse poverty situation. It is found that the average age of the older adults in the panel sample is 70.5 years old, only 14% of them are able to work, and 45.3% of the old adults are in poor households. Statistical MRPI differences exist between the older adults who participate in the HPAP and those who do not participate in any variable except labor ability. Moreover, 49.0% of the old adults participate in the farmers' cooperative programs, which is the highest participation rate compared with other programs. The IV of distance indicates that the majority of the older adults who participate in the HPAP live close to the national road.

### Regression results

Table 5 shows the OLS regression result for the determinants of the MRPI and the treatment effect model regression for the policy evaluation for the second stage.

**Table 5:** Determinants of the MRPI and the impact of the HPAP on the MRPI.

**Variables**	**The OLS result for MRPI**		**The second stage for MRPI**	
	**Coef**.	**Std. Err**.	**Coef**.	**Std. Err**.
**HPAP**			−0.159^***^	(0.004)
**Characteristics of the older adults**				
Gender	0.002	(0.001)	−0.003	(0.002)
Age	0.000	(0.000)	0.000^*^	(0.000)
Ability to work	−0.033^***^	(0.003)	−0.023^***^	(0.004)
**Family characteristics**				
Poor household	0.028^***^	(0.004)	0.001	(0.008)
Family size	−0.054^***^	(0.003)	−0.060^***^	(0.003)
Number of patients	0.037^***^	(0.002)	0.045^***^	(0.002)
Number of children	0.017^***^	(0.005)	0.017^**^	(0.007)
Number of students	0.041^***^	(0.003)	0.050^***^	(0.005)
Cultivated land	−0.014^***^	(0.002)	−0.010^***^	(0.002)
Fruit land	−0.003	(0.003)	−0.008	(0.005)
**Year effects**				
2015	0.041^***^	(0.007)		
2016	0.079^***^	(0.006)	0.039^***^	(0.004)
2017	0.070^***^	(0.005)	0.025^***^	(0.005)
2018	0.126^***^	(0.010)	0.077^***^	(0.007)
2019	0.272^***^	(0.011)	0.208^***^	(0.012)
2020	0.061^***^	(0.006)	0.024^**^	(0.009)
**Other poverty alleviation policies**				
Social security guarantees			0.025^***^	(0.005)
Farmers' cooperatives			−0.011	(0.008)
Industrial poverty alleviation			0.071^***^	(0.014)
Relocation of migrants			0.017	(0.012)
Constant	0.189^***^	(0.017)	0.302^***^	(0.015)
Town	Fixed		Fixed	
Observations	83,350		65,787	
*R* Square	0.465			
Instrumental variables		Distance and poor village		
First-stage *F*-statistic		8.78		
Overidentifying restrictions *J*-test and *p*-value		44 (0.000)		

*To control the endogeneity of the policy variables, we use the lag term of the policy variables, so the sample size is reduced to 65,787. The robust standard errors are presented in parentheses*.

*** *p < 0.01*,

** *p < 0.05*,

* *p < 0.1*.

In the OLS result of the MRPI for pooled cross-sectional data from 2014 to 2020, we find no evidence for gender and age differences, but the statistical significance is shown for all the other control variables, including demographic, family characteristics, and township and year effect. Especially, older adults without work ability are more likely to experience MRP, and the human resources, such as family size, and natural assets, such as cultivated land, contribute to the downward trend of the MRPI. However, family burdens, including the presence of patients, children, and students in the family, definitely increase the possibility of MRP among older adults. Although from 2014 to 2020, absolute income poverty has been eliminated, the positive year dummy coefficients show the increasing effect on the MRPI compared to the base year by controlling for other independent variables.

In the second-stage treatment effect model, the HPAP coefficient is significantly negative, indicating that participation in the HPAP can significantly reduce the MRPI of the old adults. Among the other policy control variables, only farmers' cooperatives have the expected poverty alleviation effect, but it is not significant. We control for the MRPI determinants and also the year and township, and this shows an identical result with the benchmark model. The first-stage F-statistic and the over-identification test of the J-statistics are also shown in Table 5.

### Analysis of heterogeneity

In J County, the HPAP includes seven types of measures: public health service, reimbursement of treatment expenses, incentive assistance, basic medical insurance, serious illness insurance, medical aid, and treatment of serious and endemic diseases. Have all of these measures been effective in reducing the MRPI? Which one is more effective? We introduce dummy variables for seven HPAP measures to explore these issues and control for endogeneity through the lag phase method. The results are shown in Panel A in Table 6. The regression results show evidence that basic health insurance and treatment of serious and endemic illnesses are effective in mitigating MRP in older adults, while the other measures are not. The three measures, public health service, incentive assistance, and medical aid, were not statistically significant. Serious illness insurance and medical expense reimbursement widened the MRPI gap in older adults to varying degrees. The result is expected since, although it is understandable that all anti-poverty programs reduce absolute poverty or minimize capability deprivations, it is possible to find that some of them increase relative poverty because they tend to help relatively the better-off more. In the case of health insurance, for example, basic health insurance covers the entire population and therefore reduces relative poverty, while serious illness insurance is affordable only to wealthier seniors and therefore increases relative poverty. The HPAP alleviates MRP in older adults by improving their health status. Will its effects differ for older adults with different levels of health? We introduce the dummy variables of serious disease, chronic disease, and disability, as well as their intersection with the HPAP to test this hypothesis. The results are shown in Panels B to D in Table 6.

**Table 6:** Analysis of heterogeneity.

**Variables**	**Coef**.	**Std. Err**.
**Panel A: Basic regression model** **+** **Health poverty alleviation measures**		
HPAP	−0.160^***^	(0.004)
L.Public health service	0.005	(0.005)
L.Medical expense reimbursement	0.007^***^	(0.002)
L.Incentive assistance	0.004	(0.003)
L.Basic medical insurance	−0.053^**^	(0.018)
L.Serious illness insurance	0.056^**^	(0.020)
L.Medical aid	0.003	(0.004)
L.Treatment of serious and endemic diseases	−0.013^*^	(0.006)
**Panel B: Basic regression model** **+** **Serious ill** +Interaction term (serious ill*HPAP)		
HPAP	−0.159^***^	(0.004)
L.serious ill	−0.001	(0.008)
L.serious ill * HPAP	0.002	(0.011)
**Panel C: Basic regression model** **+** **Chronic ill** +Interaction term (chronic ill*HPAP)		
HPAP	−0.163^***^	(0.005)
L.chronic ill	−0.012^***^	(0.003)
L.chronic ill * HPAP	0.013^***^	(0.004)
**Panel D: Basic regression model** **+** **Disability** +Interaction term (disability*HPAP)		
HPAP	−0.145^***^	(0.004)
L.disability	0.098^***^	(0.004)
L.disability * HPAP	0.036^***^	(0.002)
**Panel E: Basic regression model** **+** **Poverty caused by illness** +Interaction term (Poverty caused by illness*HPAP)		
HPAP	−0.159^***^	(0.006)
Poverty caused by illness	−0.02^***^	(0.004)
Poverty caused by illness*HPAP	0.004	(0.005)

*All the model samples were 65,787 and passed the Wald Test. The other control variables and the regression results of the selection model are as the same as the benchmark model in Table 5. The meanings of MRPI and HPAP are the same as those in Table 5. The robust standard errors are in parentheses*.

*** *p < 0.01*,

** *p < 0.05*,

* *p < 0.1*.

In Panel B, the partial effect on the MRPI for older adults with serious illness who participated in the HPAP was −0.157 (=-0.159+0.002), which was lower than that of the seriously ill older adults who did not participate (−0.001). Similarly, as shown in Panels C and D, the coefficients for older adults with chronic illness and disability who participated in the HPAP were −0.162 and −0.011, respectively, which were lower than those older adults with chronic illness and disability who did not participate in the HPAP (−0.012 and 0.098). In conclusion, older adults with chronic illnesses or disabilities can benefit from the HPAP.

Among all the poor older adults, those who are poor due to illness account for the largest share of the total poor population, ~39.89%. We further explore the effect of the policy on those who are poor due to illness. The results are presented in Panel E in Table 6. We can find that the coefficient for the people who were poor due to illness and participated in the HPAP was −0.175, which was lower than that of the seriously ill older adults who did not participate (−0.02). This result is consistent with those presented in Panels B to D in Table 6.

## Discussion

Our current research provides new evidence for MRP in older adults. Although poverty research has been well-developed in two paths, this study examines poverty among older adults from both multidimensional and relative perspectives for the first time. On the one hand, a single income or consumption cannot capture the poverty of the old adults accurately (70), and the poverty among older adults can be summarized into four dimensions: health, social, mental, and material dimensions. On the other hand, older adults are disadvantaged in income, health, and cognition, making them relatively poor (59), and the vulnerability of the older people compared with the whole population is obvious, instead, the study of individual relative poverty compared with the seniors group itself is of more theoretical and practical significance.

Our study found that the MRPI of rural older adults increased from 2014 to 2020, although all of them were lifted out of absolute income poverty by the end of this period. The health dimension accounts for about 30% of the MRPI among older adults (42). As they age, the health of older adults deteriorates (59). This reduces their source of income and increases their health care burden, pushing them into poverty. To alleviate the MRP faced by the older people, it is necessary to improve the health security system as well as pensions and health insurance for rural older adults.

We empirically examined the determinants of the MRP among older adults in the benchmark model. We find that older adults without work ability are more likely to experience MRP, while natural assets like cultivated land help lower the MRP situation. This is because, in rural areas, the main occupation for older people is agricultural work. If a senior loses the ability to work because of a disability, he or she loses his or her main economic income and increases his or her corresponding financial burden of health care. Conversely, if there are more natural assets, more agricultural income can be earned. The larger the family size of rural older adults, the less likely they are to fall into poverty. A larger family size means a higher proportion of the workforce, which indicates that rural older adults are able to receive more intergeneration financial support from family members. The corresponding family burdens, including patients, children, and students in the family, definitely increase the possibility of MRP among older adults. The positive year dummy coefficients show the increasing effect on MRP by controlling for other independent variables. This indicates that the MRP among older adults keeps increasing compared with the base year of 2014.

We further investigated the impact of the HPAP on the MRP among older adults. The results show that the HPAP can significantly alleviate MRP in older adults. In particular, basic health insurance has the most obvious effect on poverty reduction, and these two measures (medical expense reimbursement and serious illness insurance) will widen MRP. This may be because of the following reasons: first, in the social security system, basic medical insurance is the program that benefits the widest range of people, and it requires a substantial amount of funds. Moreover, there are additional financial subsidies for family members in special hardship conditions, such as minimum living security recipients. This measure has a greater impact on the overall effect of income redistribution in the social security system, while other health poverty alleviation measures do not have redistributive characteristics. In addition, according to the samples, it is found that non-poor older adults are the majority of the beneficiaries of these measures. For example, 9,885 non-poor older adults receive public health services, while only 404 poor older adults benefit from this poverty alleviation policy, which also further increases the overall inequality among older adults.

The HPAP can effectively alleviate MRP among older adults with chronic diseases or disabilities. Poverty due to illness and return to poverty due to illness are more common in rural areas. In the sample of this study, for example, ~39.89% of the older adults were poor due to illness and 47.56% returned to poverty due to illness. The HPAP can alleviate MRP among older adults, especially for those whose poverty is caused by chronic diseases and disablement. This finding shows the significant contribution of the HPAP to help to support rural older adults in the long run.

## Conclusion and policy implication

By constructing the MRPI of older adults, this study finds that relative poverty still persists for rural older adults, showing an upward trend. In addition, among the four MRPI dimensions, the health component plays the most important role rather than mental, social, and material factors for older adults. Using the Poverty Alleviation Database of J County in Shaanxi Province from 2014 to 2020, we empirically show the determinants of the MRPI, and we find that the HPAP can significantly alleviate MRP among older adults. In particular, basic health insurance has the most evident effect on poverty reduction. The HPAP can effectively alleviate MRP among older adults with chronic diseases or disabilities. Therefore, it is important to emphasize that the focus should be on vulnerable populations when implementing pro-poor policies. Improving the quality of health management and services is an effective way to block the occurrence of MRP in rural older adults. Presently, in China, constructing an integrated health promotion system is necessary and imminent. Specifically, popularizing health knowledge for all ages, establishing hierarchical diagnosis and treatment systems, conceptualizing medical insurance systems, such as long-term care insurance, and providing medical integration will efficiently reduce relative poverty in the future.

### Strength and limitations

Our study has various strengths and limitations. A significant strength is that we try to construct the MRPI by combining the multidimensional and relative issues to analyze poverty among older adults. This provides an essential reference point for further studies on relative poverty among older adults.

Although the fuzzy set approach can avoid the disadvantages of arbitrary equal weights and the inability to measure the relativity better than the AF method, it has apparent drawbacks, for example, the weights cannot be fixed in different years. Recent solutions regarding data-driven endogenous weights for calculating the MRPI are provided by Dutta et al. (71), but the application of panel data or pooled cross-sectional data as well as selecting the base period or full period data will still ensue different weights, resulting in cross-period incompatibility. Therefore, the possibility of potential expansion still exists.

Additionally, limited to the availability of data, we focus only on older adults at the edge of the poverty line. This might underestimate the MRP incidence among the entire aged population. There is also a lack of subjective indicators in the mental dimension (72), instead, we choose proxy variables which can cause the possibility of imprecision. This article may also suffer from the problem of omitting variables (e.g., the ability, personality, and preferences of the elderly).

## Data availability statement

The dataset is administrative data, not open to public use so far. Requests to access these datasets should be directed to zengwh@mail.xjtu.edu.cn.

## Author contributions

WZ conceived the idea. PZ and YZ participated in data collection and statistical analysis. WZ and PZ drafted the manuscript and edited the paper. RS gave many valuable comments on the draft and polished it. All the authors contributed toward revising the manuscript and have read and approved the final manuscript.

## Funding

This study was supported by the two Social Sciences Funds of Shaanxi Province in China under Grant numbers 2020D004 and 2019D014.

## Conflict of interest

The authors declare that the research was conducted in the absence of any commercial or financial relationships that could be construed as a potential conflict of interest.

## Publisher's note

All claims expressed in this article are solely those of the authors and do not necessarily represent those of their affiliated organizations, or those of the publisher, the editors and the reviewers. Any product that may be evaluated in this article, or claim that may be made by its manufacturer, is not guaranteed or endorsed by the publisher.

## Footnotes

^1^Poverty-stricken counties exit criteria: First, the combined poverty incidence rate is reduced to <2%. Second, all poor villages are out of poverty. Third, the growth rate of per capita disposable income of rural residents is higher than the average level of the province. Fourth, the level of basic public services reached or got close to the provincial average.

^2^Stata command: svyset town [pweight = finalwgt]; svy: reg MRPI *X*_*it*_ i.year i.town.
