# Supplementary material for: The multidimensional relative poverty of rural older adults in China and the effect of the health poverty alleviation policy
Source: Front Public Health. 2022 Jul 22;10:793673. doi: 10.3389/fpubh.2022.793673 (PMC9354235; doi:10.3389/fpubh.2022.793673)
Supplement: Supplementary file 1 [file Table_1.DOCX]

**Appendix A**

**Table A The mean and calculated weights (in parentheses) for indicators of four dimensions from 2014 to 2020**

| **Dimensions** | **Indicators** | **2014** | **2015** | **2016** | **2017** | **2018** | **2019** | **2020** |
| --- | --- | --- | --- | --- | --- | --- | --- | --- |
| **Dim_1_: Health** | **Disability** | 0.136 | 0.137 | 0.134 | 0.138 | 0.132 | 0.172 | 0.180 |
|  |  | (1.998) | (1.991) | (2.009) | (1.981) | (2.028) | (1.759) | (1.716) |
|  | **Disease** | 0.465 | 0.480 | 0.496 | 0.428 | 0.389 | 0.462 | 0.496 |
|  |  | (0.766) | (0.734) | (0.702) | (0.848) | (0.944) | (0.773) | (0.701) |
|  | **No Health insurance** | 1.000 | 1.000 | 1.000 | 1.000 | 0.998 | 0.992 | 0.992 |
|  |  | (0) | (0) | (0) | (0) | (0.002) | (0.008) | (0.008) |
| **Dim_2_: Social** | **No Work** | 0.985 | 0.976 | 0.965 | 0.942 | 0.849 | 0.868 | 0.846 |
|  |  | (0.015) | (0.024) | (0.036) | (0.059) | (0.164) | (0.142) | (0.167) |
|  | **No Pension** | 0.050 | 0.058 | 0.056 | 0.054 | 0.008 | 0.000 | 0.000 |
|  |  | (3) | (2.855) | (2.89) | (2.916) | (4.789) | (0) | (9.585) |
|  | **No Party member** | 0.983 | 0.980 | 0.970 | 0.961 | 0.959 | 0.948 | 0.949 |
|  |  | (0.017) | (0.021) | (0.031) | (0.04) | (0.042) | (0.054) | (0.052) |
|  | **No Radio or TV** | 0.792 | 0.785 | 0.767 | 0.745 | 0.414 | 0.273 | 0.269 |
|  |  | (0.233) | (0.241) | (0.265) | (0.295) | (0.881) | (1.298) | (1.312) |
| **Dim_3_: Mental** | **Education** | 0.887 | 0.912 | 0.945 | 0.970 | 0.974 | 1.004 | 1.033 |
|  |  | (0.251) | (0.259) | (0.209) | (0.216) | (0.217) | (0.224) | (0.231) |
|  | **Live alone** | 0.307 | 0.299 | 0.279 | 0.256 | 0.231 | 0.232 | 0.233 |
|  |  | (1.182) | (1.207) | (1.278) | (1.361) | (1.467) | (1.459) | (1.456) |
| **Dim_4_: Material** | **Per capita income** | 2353 | 2879 | 4120 | 5005 | 6225 | 7114 | 8420 |
|  |  | (0.116) | (0.569) | (0.223) | (0.178) | (0.132) | (0.14) | (0.05) |
|  | **Per capita housing area** | 28.368 | 29.006 | 31.627 | 30.440 | 29.629 | 29.956 | 30.259 |
|  |  | (0.048) | (0.046) | (0.011) | (0.107) | (0.137) | (0.154) | (0.161) |
|  | **No Fuel** | 1.000 | 1.000 | 1.000 | 1.000 | 0.999 | 0.998 | 0.978 |
|  |  | (0) | (0) | (0) | (0) | (0.001) | (0.002) | (0.022) |
|  | **No Electricity** | 0.025 | 0.017 | 0.000 | 0.000 | 0.000 | 0.000 | 0.000 |
|  |  | (3.692) | (4.075) | (0) | (0) | (0) | (0) | (0) |
|  | **No Safe drinking water** | 0.147 | 0.057 | 0.030 | 0.007 | 0.000 | 0.000 | 0.000 |
|  |  | (1.921) | (2.855) | (3.506) | (4.936) | (0) | (0) | (0) |
|  | **No Sanitary toilet** | 0.019 | 0.298 | 0.525 | 0.624 | 0.560 | 0.580 | 0.560 |
|  |  | (3.954) | (1.213) | (0.645) | (0.471) | (0.58) | (0.544) | (0.579) |

Note: The weights are in parentheses.
